# Elevated Levels of Soluble CTLA-4, PD-1, PD-L1, LAG-3 and TIM-3 and Systemic Inflammatory Stress as Potential Contributors to Immune Suppression and Generalized Tumorigenesis in a Cohort of South African Xeroderma Pigmentosum Patients

**DOI:** 10.3389/fonc.2022.819790

**Published:** 2022-02-11

**Authors:** Mahlatse C. M. Kgokolo, Katherine Anderson, Shalate C. Siwele, Helen C. Steel, Luyanda L. I. Kwofie, Mike M. Sathekge, Pieter W. A. Meyer, Bernardo L. Rapoport, Ronald Anderson

**Affiliations:** ^1^ Department of Dermatology, Faculty of Health Sciences, University of Pretoria and Steve Biko Academic Hospital, Pretoria, South Africa; ^2^ Department of Immunology, School of Medicine, Faculty of Health Sciences, University of Pretoria, Pretoria, South Africa; ^3^ Tshwane Academic Division of the National Health Laboratory Service, Pretoria, South Africa; ^4^ Department of Nuclear Medicine, Faculty of Nuclear Medicine, Faculty of Health Sciences, University of Pretoria and Steve Biko Academic Hospital, Pretoria, South Africa; ^5^ The Medical Oncology Centre of Rosebank, Johannesburg, South Africa

**Keywords:** C-reactive protein, cutaneous malignancies, cytokines, DNA excision/repair, 8-hydroxy-2-deoxyguanosine, soluble inhibitory immune checkpoints, programmed death protein-1 (PD-1), vitamin D

## Abstract

Xeroderma Pigmentosum (XP), an autosomal recessive disorder characterized by ultraviolet radiation-induced abnormalities of DNA excision and repair pathways is associated with early development of cutaneous cancers. Intracellular oxidative stress has also been proposed as a contributor to the occurrence of skin cancers. However, little is known about the possible augmentative contributions of chronic inflammation, immune suppression and oxidative stress to the pathogenesis of malignancies associated with other subtypes of XP. This has been addressed in the current study, focused on the measurement of systemic biomarkers of inflammation, immune dysfunction and oxidative damage in XP patients, consisting of XP-C, XP-D and XP-E cases, including those XP-C cases who had already developed multiple skin malignancies. The inflammatory biomarker profile measured in XP patients and healthy control subjects included the cytokines, interleukins (ILs)-2, -4, -6, -10, interferon-γ (IFN- γ) and tumor-necrosis factor-α (TNF-α), the acute phase reactant, C-reactive protein (CRP), and cotinine (as an objective indicator of smoking status). Immune suppression was detected according to the levels of five soluble inhibitory immune checkpoint proteins (CTLA-4, PD-1, PD-L1, LAG-3 and TIM-3), as well as those of vitamin D, while oxidative stress was determined according to the circulating levels of the DNA adduct, 8-hydroxy-2-deoxyguanosine (8-OH-dG). These various biomarkers were measured in plasma using immunofluorimetric, nephelometric and ELISA procedures. Significant elevations in IL-6 (*P*<0.01) and TNF-α (*P*<0.0001), but none of the other cytokines, as well as increased levels of all five soluble inhibitory immune checkpoints (*P*=0.032-*P*=0.0001) were detected in the plasma of the XP patients. C-reactive protein and vitamin D were increased and decreased, respectively (both *P*<0.0001), while only one participant had an elevated level of plasma cotinine. Surprisingly, the levels of 8-OH-dG were significantly (*P*=0.0001) lower in the group of XP patients relative to a group of healthy control subjects. The findings of increased levels of pro-inflammatory cytokines and, in particular, those of the soluble immune checkpoints, in the setting of decreased vitamin D and moderately elevated levels of CRP in XP patients suggest a possible secondary role of ongoing, inflammatory stress and immune suppression in the pathogenesis of XP-associated malignancies.

## Introduction

Xeroderma pigmentosum (XP) is an autosomal recessive, genetic disorder associated with clinical and cellular hypersensitivity to ultraviolet (UV) radiation and defective repair of DNA, which is characterized by early development of cutaneous and ocular malignancies. Xeroderma pigmentosum occurs worldwide, affecting all races with no sex predilection. It is subdivided into eight complementation groups: XP-A to XP-G and XP variant (XP-V). The prevalence of XP is 1:1,000,000 in the United States and Europe and is 1:100,000 in Japan. It is more common in populations where marriage of close blood relatives is common. In addition to having a major predisposition for development of cutaneous cancers, XP patients also have an approximately 10-to-20-fold increase in the incidence of internal malignancies, including tumors of the brain, spinal cord, lung, uterus, breast, pancreas, stomach, gastrointestinal tract, kidney and hematopoietic system (1, 2). As these areas are not sun-exposed, chronic inflammation, immune suppression and oxidative stress may represent additional, possible contributors to the development of cancer in XP.

Inflammatory responses, both acute and especially chronic, are strongly associated with increased levels of oxidative stress, due in large part to excessive and/or poorly controlled release of reactive oxygen species (ROS) by activated cells of the innate immune system, particularly phagocytes, as well as by exposure to cigarette smoke, which can be detected by measurement of the nicotine metabolite, cotinine Indeed, the association between chronic inflammation and development of epithelial cancers has been recognized for over a century (3). In this context, inflammatory processes can both initiate and drive tumorigenesis *via* various mechanisms. These include the production of growth factors that sustain proliferative signaling, survival factors that attenuate apoptosis and pro-angiogenic factors such as vascular endothelial growth factor (VEGF) and extracellular matrix-modifying enzymes that enable angiogenesis, invasion, and metastasis, as well as inductive signals that lead to activation of epithelial-mesenchymal transition (EMT) and other oncogenic processes (4). Additionally, cancer can evade protective anti-tumor T cell-mediated immune responses by upregulating expression of inhibitory immune checkpoint molecules (5).

In this context, it is noteworthy that circulating levels of the acute phase reactant, C-reactive protein (CRP), have been found to be moderately elevated in patients with various types of cancer (6, 7). Indeed, epidemiologic data suggest that elevated levels of CRP, when measured by high-sensitivity assays, are not merely a marker of prevalent cancer, but are also associated with an increased risk of future cancer in apparently healthy individuals (8–14). This contention is supported by the findings of two large prospective cohort studies that described positive associations between elevated circulating levels of CRP and the risk of cancer of any type (9, 11).

Persistently elevated levels of CRP also appear to be predictive of poor responses to therapy in patients with certain types of malignancy. This scenario is exemplified by the findings of a study focused on the relationship between elevated concentrations of circulating CRP and overall survival in prostate cancer patients receiving specific chemotherapy (docetaxel-based) (15). The aforementioned findings are also supported by a number of more recent studies, including those in patients with pancreatic neuroendocrine neoplasms, oral squamous cell carcinoma, non-small cell lung carcinoma, and possibly “most adult solid tumors” (16–19). In addition, circulating levels of CRP have been reported to be “markedly deranged” in patients with incurable cancer approaching death (20). Although not entirely clear, CRP-mediated inhibition of the activation and proliferation of anti-tumor cytotoxic T cells may underpin the pro-tumorigenic activity of this acute phase protein (21).

Cytokines/chemokines are also key players in the immunopathogenesis of cancer, both by activating and sustaining acute and chronic inflammatory responses. Although mostly beneficial, if poorly regulated, production of cytokines during chronic inflammatory disorders of both infective and non-infective origin may predispose for development of cancer. In this context, the pro-inflammatory cytokines, tumor necrosis factor-α (TNF-α) and interleukin (IL)-6 (a major inducer of CRP), as well as the immunosuppressive cytokine, IL-10, have all been implicated in both the initiation and development of cancer (22).

More recently, the key role played by inhibitory immune checkpoint molecules in suppressing anti-tumor immune responses has not only underscored the critical involvement of the human immune system in controlling the development and spread of cancer, but has also revitalized the practice of anti-cancer immunotherapy (23, 24). Inhibitory immune checkpoints, which are produced by suppressor cells of lymphoid and myeloid origin, as well as by tumor cells and structural cells in the tumor microenvironment (TME), interact with counter receptors on various types of immune effector cells such as cytotoxic T cells, natural killer cells and antigen-presenting cells, especially dendritic cells (23, 24). Prominent examples include: i) cytotoxic T lymphocyte-associated antigen-4 (CTLA-4, CD152); ii) programmed cell death protein 1(PD-1, CD279) and its ligand, PD-L1 (CD274); iii) lymphocyte-activation gene-3 (LAG-3, CD233) and iv) T-cell immunoglobulin and mucin-domain containing-3 (TIM-3, CD366). To our knowledge, however, little is known about the expression levels of these inhibitory immune checkpoints, either systemically or in the TME, of patients with XP. Of note, however, are a series of case reports, albeit limited, of patients with XP, which describe favorable responses, mostly in the clinical settings of metastatic melanoma and metastatic squamous cell carcinoma, following treatment with PD-1-targeted monoclonal antibodies, most commonly pembrolizumab (reviewed in 25, 26).

With respect to plasma vitamin D status, a high prevalence of deficiency of this vitamin has been reported in young (6-25 years) Japanese patients with the XP-A variant of XP (25), probably due to efficient implementation of photo-protective measures. Although seemingly unexplored in the setting of XP, it is noteworthy that according to a secondary analysis of data from the “Vitamin D and Omega-3 Trial (VITAL)” to which cancer-free, older, (≥50-55 years) adult humans were recruited (*n*=25,871), vitamin D supplementation was found to reduce the incidence of both metastatic and fatal cancer (26). While various mechanisms may contribute to the apparent anti-cancer properties of vitamin D, it is noteworthy that in another study vitamin D supplementation of women with established breast cancer was found to attenuate tumor growth, which was associated with decreased inflammation and increased numbers of tumor-infiltrating CD8^+^ cytotoxic T cells (27).

Currently, little is known about the possible coexistence of systemic inflammation, immune suppression and oxidative stress in patients with XP. This issue has been addressed in the current study in which the systemic levels of various biomarkers of oxidative [8-hydroxy-2-deoxyguanosine (8-OH-dG) and cotinine] and inflammatory/immunosuppressive stress [CRP, IL-2, IL-4, IL-6, IL-10, Interferon-γ (IFN-γ), tumor necrosis factor- α (TNF-α)], as well as immunosuppression (inhibitory immune checkpoints and vitamin D) have been measured in an unusually large cohort (*n*=24) of patients with XP.

## Patients

The study population consisted of a total of 23 South African XP patients attending the Dermatology Screening Clinic at Steve Biko Academic Hospital, Pretoria, South Africa. Measurements of plasma cytokines, CRP, 8-OH-dG and cotinine were performed on 19 of these patients of whom 17 were black (9F:8M) and two white (both F). The ages of the black patients varied between three years to nineteen years with 73.7% (*n*=14) aged between 3 and 10 years and 15.8% (*n*=3) aged 16-19 years. The remaining patients (*n*=2, 10.5%) were two white adult sisters aged 51 and 48 years. As shown in **Table 1**, all patients presented with typical dermatological and ophthalmic features of XP. None of the parents had clinical XP disease. Two black families and the white family reported one sibling affected by XP who died from XP-related disease complications. Measurements of plasma soluble inhibitory immune checkpoints and vitamin D were performed on 15 black South African XP patients, 11 from the aforementioned group, together with an additional 4 subsequently recruited patients (numbers 20-23) on whom the diagnosis of XP was based on clinical criteria (1, 28, 29). The ages of this cohort of XP patients ranged from 2 to 9 years with the demographic and clinical data of the additional patients also shown in **Table 1**.

**Table 1:** Phenotypic and genotypic characteristics of XP patients.

		Age		Clinical manifestations		
		onset	current	skin	mouth	eye involvement
*XPC families*						
03	F	18 m	19 y	BCC SCC	tongue/lip	keratopathy/tumor
04	F	6 m	18 y	BCC SCC	tongue	enucleation/tumor
06	F	9 m	10 y	BCC SCC	tongue	corneal scarring
08	M	2 y	6 y	BCC SCC	tongue	keratopathy
10	M	2 y	8 y	BCC SCC	tongue	corneal scarring
11	F	3 y	16 y	BCC SCC	tongue	corneal scarring
12	F	2 y	8 y	BCC SCC	tongue/lip	keratopathy
14	M	–	3 y	none	none	photophobia
15	M	1 y	10 y	BCC SCC	tongue	corneal scarring
16 & 17	M	2 y	9 y	BCC SCC	tongue	keratoconjunctivitis
18	F	1 y	6 y	BCC SCC	None	fibrosis
19	F	9 m	6 y	BCC SCC	lip	eyelid tumor
*XPD families*						
05	M	–	4 y	freckling/sun sensitivity	none	keratopathy/enucleation
09	F	–	10 y	freckling/sun sensitivity	none	photophobia/MR
*XPE family*						
01	F	–	51 y	freckling/sun sensitivity	none	none
02	F	–	48 y	freckling/sun sensitivity	none	none
*Other families*						
07	F	Birth	4 y	freckling/skin xerosis	none	none
13	F	–	6 y	freckling	none	none
20	M	2 y	5 y	SCC	tongue/lip	photophobia
21	F	8 m	2 y	SCC	none	keratopathy/tumor/corneal scarring
22	M	3 y	9 y	SCC	tongue/lip	tumor/photophobia
23	M	3 y	7 y	freckling/actinic keratosis	Actinic cheilitis	photophobia

F, female; M, male; m, month; y, year; BCC, basal cell carcinoma; SCC, squamous cell carcinoma; MR, mental retardation.

All patients were recruited over a period of 5 years. Informed consent was obtained from all the adult patients and from the parents and guardians of the affected children, as well as from the healthy control participants mentioned below, all of whom fully understood the purpose of the study, which was undertaken in full compliance with the 1964 Declaration of Helsinki. The genetic studies that were performed on the XP patients to accurately define genotype have recently been described elsewhere (30).

Ethics approval was granted by The Research Ethics Committee, Faculty of Health Sciences, University of Pretoria (Ethics Committee Approval Numbers 326/2016, 251/2019 and 510/2020). Twelve black South African healthy control subjects [6 non-smokers (all F; mean age 30.3 ± 5.5 years) and six smokers (1F:5M; mean age 29.2 ± 4.3 years as a positive control group for oxidative stress)] were included in the analysis of 8-hydroxy-2-deoxyguanosine (8-OH-dG). For the cytokine analyses, a group of 15 healthy, non-smoking black South African subjects (9F:6M; mean age 32.5 ± 4.8 years) was included for comparison. The control group for the immune checkpoint study consisted of five black South African control subjects (3F:2M; mean age 25.0 ± 6.3 years). Controls for the vitamin D study included an additional two white control subjects (both female; mean age 53 ± 7.1 years).

## Methods

Whole venous blood samples were collected in ethylenediaminetetraacetic acid (EDTA) vacutainers on different dates in two batches of varying sizes during September 2019 and November 2020 and processed promptly to separate the plasma component by centrifugation and stored at minus 70°C. Plasma was used as the matrix for the various laboratory procedures described below.

### Measurement of 8-Hydroxy-2-Deoxyguanosine (8-OH-dG)

This was performed using a commercial competitive enzyme-linked immunosorbent assay (ELISA) procedure (DNA/RNA Oxidative Damage ELISA kit) supplied by the Cayman Chemical Company, Ann Arbor, MI, USA. These results are expressed as picograms (pg) 8-OH-dG per milliliter (mL) plasma with data analysis performed using data reduction software, which plots the data automatically.

### Cytokine Analysis

These were measured using the Bio-Plex^®^ suspension bead array system (Bio-Rad Laboratories Inc., Hercules, CA, USA), which utilizes Luminex xMAP multiplex technology to enable simultaneous detection and quantitation of multiple different analytes in a single sample. The following analytes were measured using this system: IL-2, IL-4, IL-6, IL-10, TNF-α and IFN-γ; these cytokines were considered to be representative of Th1 cells, Th2 cells and monocytes/macrophages. A wide range of standards (0.38-91756.00 pg/mL) was used to enable quantitation of the individual cytokines using a Bio-Plex^®^ array reader with a dual detector and real-time digital signal processing. The results are expressed as pg/mL plasma for each of the test cytokines.

### C-Reactive Protein

Plasma CRP was assayed using high-sensitivity laser nephelometry (Siemens Healthcare Diagnostics, Atellica NEPH 630 Nephelometer, Newark, NJ, USA). The results are expressed as micrograms (µg)/mL plasma with values of >2.5 µg/mL considered to be elevated.

### Soluble Inhibitory Immune Checkpoints

A Human Immuno-Oncology Checkpoint Protein Panel (Milliplex™ MAP Kit, Merck, KGaA, Darmstadt, Germany) was used to simultaneously determine the plasma concentrations of five soluble inhibitory immune checkpoint proteins, namely CTLA-4, PD-1, PD-L1, LAG-3 and TIM-3, using suspension bead array technology in microplate format. The methodology was performed as per the manufacturer’s instructions. The plate was analysed using a Bio-Plex Suspension Array platform using the Bio-Plex Manager software 6.0 for bead acquisition and measurement of median fluorescence intensity. The results are reported as the plasma concentrations (pg/mL) of each of the five test inhibitory immune checkpoints.

### Vitamin D

Plasma concentrations of vitamin D were measured using the Euroimmun 25-OH Vitamin D ELISA procedure according to the manufacturer’s instructions (Euroimmun Medical Laboratory Diagnostics G, Lübeck, Germany). This is a competitive capture ELISA procedure in which 25-OH vitamin D in plasma (diluted 1:26) competes with added biotin-labeled 25-OH vitamin D for binding to the Fab sites of a 25-OH vitamin D-specific monoclonal antibody adsorbed to the wells of a 96-well microplate. Detection of the amount of antibody-bound biotin-labeled 25-OH involves successive addition of peroxidase-labeled streptavidin and a colorimetric substrate. Color intensity detected spectrophotometrically at 450 nm is inversely proportional to the concentration of 25-OH vitamin D in the test plasma samples, which is expressed as nanograms (ng)/mL calculated from a standard curve constructed from known standards.

### Cotinine

Plasma cotinine levels, as an objective indicator of smoking status, were measured using a commercial ELISA system (Calbiotech Inc., Spring Valley, CA, USA) and the results expressed as ng/mL plasma. A cut-off value of >14 ng/mL was taken as being indicative of active smoking in accordance with the manufacturer’s recommendation.

### Expression and Statistical Analysis of Results

The results of each investigation are presented as the median values and interquartile ranges (IQRs) and the Mann-Whitney *U*-test was used for comparison of non-parametric data where appropriate.

## Results

### Plasma 8-Hydroxy-2-Deoxyguanosine (8-OH-dG)

These results are shown in **Table 2**. No statistically significant differences were detected with respect to comparison of the control groups of healthy non-smokers and smokers. However, and somewhat surprisingly, plasma levels of 8-OH-dG were significantly (*P*=0.0001) lower in the cohort of the 19 XP patients tested relative to the combined group (non-smokers and smokers) of healthy control subjects.

**Table 2:** Plasma concentrations of 8-hydroxy-2-deoxyguanosine (8-OH-dG) in patients with XP and sub-groups of healthy non-smoking and smoking control subjects.

Group	Plasma concentrations of 8-OH-dG (pg/mL)
XP patients (n=19)	4701.56 (4085.27-5697.42)*
Non-smoking controls (n=6)	7078.80 (5831.85-9233.74)
Smoking controls (n=6)	6996.42 (6352.05-8418.11)
Combined group of control subjects (n=12)	6996.42 (5859.97-8838.92)^+^

*Results expressed as the median values in pg/mL plasma with 25% and 75% IQRs.

^+^P<0.001 for comparison of the XP patient group with the combined group of control subjects.

### Plasma Cytokines

Interleukin-2 and IL-4 were undetectable in the plasma of all 19 XP patients tested, while IFN-γ (3.27 pg/ml) was detected in the plasma of only one patient. Of the remaining cytokines, the median values and IQRs for TNF-α, IL-6 and IL-10 were 6.83 (5.56-8.07), 2.17 (1.97-2.87) and 0.62 (0.62-1.94) pg/mL, respectively. The plasma concentrations of the former two cytokines were significantly elevated (*P*<0.0001 and *P*<0.01, respectively) relative to the corresponding values for healthy control subjects, which were 0.73 (0.73-1.0) and 0.88 (0.88-0.93) pg/mL, respectively. As mentioned in the following section, plasma levels of TNF-α and IL-6 were also higher, albeit not significantly so, in the group of XP patients with elevated levels of CRP, relative to those patients with normal levels of the acute phase reactant.

### C-Reactive Protein

Moderately elevated levels of CRP were detected in the plasma of 8/19 XP patients tested, the values being 4.37, 4.41, 5.61, 7.61, 8.48, 9.31, 9.72 and 19.7 µg/mL [8.05 (5.31-9.41) µg/mL]. The median value and IQRs for the remaining 11 patients were [0.74 (0.24-1.08) µg/mL; range: 0.16 – 2.12 µg/mL; *P*<0.0001 for comparison between the two subgroups of XP patients]. The median values for 8-OH-dG for the groups of XP patients with low and moderately elevated levels of CRP were 4314.34 (4056.03-4975.13) and 5453.58 (4514.34-7034.51) pg/mL respectively (not significantly different). A comparison of the plasma concentrations of TNF-α, IL-6 and IL-10 in the sub-groups of XP patients with normal and elevated levels of CRP is shown in **Table 3**. Increased levels of TNF-α and IL-6, but not IL-10, were evident in the sub-group of XP patients with elevated levels of CRP, but these differences did not attain statistical significance.

**Table 3:** Comparison of the plasma levels of TNF-α, IL-6 and IL-10 in the sub-groups of XP patients with normal and elevated levels of CRP.

Cytokines (pg/mL)	XP Patients	
	Elevated CRP (n=8)	Normal CRP (n=11)
TNF-α	7.66 (6.75-11.43)*	6.27 (5.56-7.11)
IL-6	2.77 (2.17-4.43)	2.02 (1.92-2.27)
IL-10	1.59 (0.62-2.03)	0.62 (0.62-0.62)

*Results expressed as the median values in pg/mL plasma with 25% and 75% IQRs.

### Soluble Immune Checkpoints

**Figure 1:**
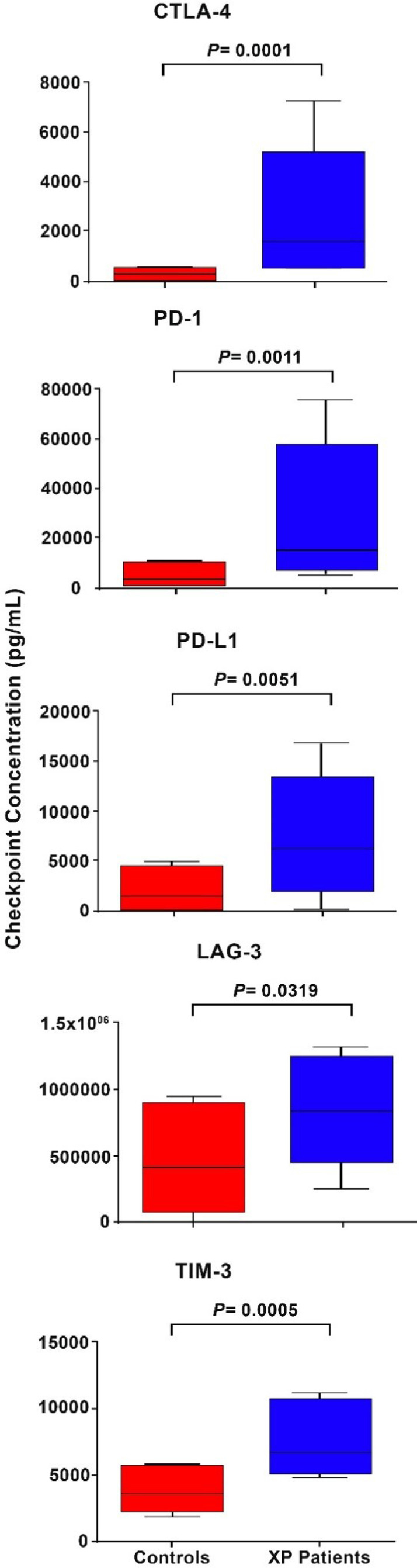
Box and whisker plots showing a comparison of the plasma concentrations as pg/mL of the five soluble inhibitory immune checkpoints, CTLA-4, PD-1, PD-L1, LAG-3 and TIM-3 in control subjects relative to those of the cohort of XP patients.

These results are shown in **Table 4** and **Figure 1**. Relative to those of the healthy control subjects, the median plasma levels of the XP patients tested (n=15) were significantly elevated, with the median fold increases of CTLA-4, PD-1, PD-L1, LAG-3 and TIM-3 being 3.7-, 2.1-, 4.6-, 1.98- and 2.47-fold (P=0.032-P=0.0001), respectively.

**Table 4:** Comparison of the concentrations of the test soluble inhibitory immune checkpoints in plasma samples from control participants and Xeroderma Pigmentosum patients.

Checkpoints	Control Participants (n=5)	Xeroderma Pigmentosum Patients (n=15)	*P* ≤
CTLA-4	418.42 (310.17–451.39)*	1550.09 (612.46–2891.79)	0.0001
PD-1	7664.25 (4364.5–9932.47)	16480.11 (11163.3–34197.63)	0.001
PD-L1	1325.81 (271.92–3665.64)	6143.29 (3954.88–9192.49)	0.005
LAG-3	423768.0 (181603.8–843313.7)	839880.1 (671236.5–1150400.0)	0.032
TIM-3	2687.71 (2594.45–3496.56)	6648.43 (5670.46–9712.61)	0.0005

*Results expressed as the median values in pg/mL plasma with 25% and 75% IQRs.

### Plasma 25-OH Vitamin D

Plasma vitamin D levels were significantly lower (*P*<0.017) in the group of XP patients tested (*n*=15) relative to those of the healthy control participants [17.3 (8.1-29.4) vs 27.8 (25.2-30.0) ng/mL]. Of the 15 XP patients, 8 had plasma vitamin D values ≤20 µg/mL [14.8 (8.85-15.43), 3 of whom were severely deficient with values in the range 5-10 µg/mL

### Plasma Cotinine as an Indicator of Cigarette Smoke Exposure

An elevated level of plasma cotinine (>50 ng/mL) was detected in one patient, but was undetectable in the plasma of all the remaining patients. The 8-OH-dG and CRP values for the XP patient who smoked were 4917.09 pg/mL and 7.61 µg/mL respectively, while the corresponding plasma concentrations of TNF-α and IL-6 were 4.55 and 1.97 pg/mL, respectively.

## Discussion

Notwithstanding an expectedly very low frequency of current smoking in the setting of unexpectedly low plasma concentrations of the oxidized purine nucleoside, 8-OH-dG, the results of the current study have identified previously undocumented systemic inflammatory/immunosuppressive changes in South African XP patients. These include increased plasma levels of the pro-inflammatory cytokines TNF-α and IL-6, and, in particular, those of the soluble inhibitory immune checkpoints, CTLA-4, PD-1, PD-L1, LAG-3 and TIM-3, together with a high prevalence (42%) of elevated levels of high-sensitivity CRP in the setting of decreased levels of vitamin D. These changes are indicative of ongoing, chronic inflammation and seemingly intense immunosuppression, which accompany, and may precede, the development of many types of cancer (31, 32). Somewhat surprisingly, the systemic concentration of immunosuppressive IL-10 was not elevated in the group of XP patients, suggesting that this cytokine may not be dysregulated in this condition. Of the 19 participants tested, 8 had raised CRP levels. Six were black participants, one of whom had moderate disease and 4 (3, 10, 12 and 18) with clinically severe disease. Of these six black participants, five were found to belong to complementation group XP-C. Of the four with clinically severe disease, patients 10 and 18, aged eight and six years, respectively, had the highest CRP levels, both having been operated on several times for excision of squamous cell carcinomas. Surprisingly though, the remaining participants, 3 and 12, aged eighteen and eight years old respectively, had lower CRP levels compared to all the others, but had also been operated on many times for excision of squamous cell carcinomas, basal cell carcinomas and a coexistent melanoma in the case of participant 12.

The second group consisted of two white participants, sisters, 1 and 2 (*n*=2). Both had clinically severe disease and belonged to complementation group E. These two participants had high levels of CRP, seemingly correlating with the severe skin cancers they had developed in their lifetime. The eldest, 51 year old participant 1, had the highest CRP level in the entire cohort. In addition to twenty squamous cell carcinomas and three basal cell carcinomas, she had developed more than 60 melanomas in her lifetime. Her younger sister, 48 year old participant 2, had developed 4 basal cell carcinomas and 8 squamous cell carcinomas.

A third group (*n*=11) of black participants had low CRP levels. Two participants, 5 and 9, presented with clinically moderate disease and were found to belong to complementation group D. These two participants were found to carry novel genes as described in a recent publication (30). However, the remaining participants (*n*=9) with low CRP levels had severe clinical disease, having been operated on several times for excision of skin cancers and other skin tumors.

With the exception of two black participants, patients 5 and 9, aged 4 and 10 years (with clinically mild and moderate disease respectively), all participants had been operated on multiple times for excision of different skin cancers (basal cell carcinoma, squamous cell carcinoma or melanoma), with all of these skin cancers occurring in one participant in the case of participant 12.

The reasons for the significantly lower plasma levels of plasma 8-OH-dG in patients with XP are somewhat difficult to explain, but may result from defective excision, leading to intracellular retention and/or modification of this oxidized purine base (33–36). Currently, there is a very limited literature on this topic, encompassing only a few patients. One study has reported elevated urinary levels of 8-OH-dG in a very young child aged 1 year and 11 months with XP/Cockayne syndrome (CS) complex (37), while another, focused on circadian rhythms, to which eight XP-A patients were recruited, reported different findings, with patients, specifically the subgroup aged <15 years, demonstrating moderately decreased levels of urinary 8-OH-dG levels relative to control subjects (38). The findings of this latter study appear to be in agreement with those of the current study and appear to question the utility of measurement of 8-OH-dG as a biomarker of oxidative stress in XP.

Nonetheless, it is important to note that oxidative damage to the nucleotide bases in DNA has been investigated previously in isolated human skin and blood cells or their cell lines from XP and CS patients, with CS cells appearing to be more sensitive to oxidative DNA lesions than XP cells (39). Exceptions included cells from XP-A patients, which exhibited defective repair of 8,5-cyclo-2-deoxyadenosine, a free radical-induced DNA lesion, while increased oxidative stress was also evident in cancer-prone XP-D fibroblasts, due to production of high amounts of superoxide and hydrogen peroxide (39). In addition, examination of brain autopsies from XP and CS patients revealed the involvement of oxidative stress in neuropathological damage (39).

In our opinion, however, the observation of greatest significance that has originated from this study is the marked increase in the plasma concentrations of the five soluble inhibitory immune checkpoint proteins, CTLA-4, PD-1, PD-L1, LAG-3, and TIM-3. Notwithstanding secretion by various types of immune suppressor cells in early premalignant lesions and the established TME, these diffusible soluble immune checkpoints are also likely to originate from various types of structural cells, such as cancer-associated fibroblasts, as well as from tumor cells *per se* (40–42). This appears to represent an effective tumor-orchestrated strategy to promote widespread systemic immunosuppression that favors tumor development and spread, reduced efficacy of anti-cancer treatment and poor prognosis (40). Such a scenario has been described in many types of solid malignancies including, but not limited to, non-small cell lung carcinoma, gastric cancer, hepatocellular carcinoma, clear cell renal carcinoma, head and neck cancer and metastatic melanoma (43–49), as well as various types of lymphoid malignancies (50–54).

In the case of XP, the very high prevalence of recurring cutaneous malignancies results from the defective excision repair of mutations that occur with a very high frequency in genes that regulate cell proliferation, especially *ras* oncogenes and tumor suppressor genes, following exposure to UV radiation (55, 56), while immunodeficiency associated with DNA repair defects may also contribute (57). Chronic tumorigenesis, in turn, is likely to potentiate tumor-mediated persistence and spread *via* establishment of a highly immunosuppressive milieu, involving production not only of soluble inhibitory immune checkpoints, but also other mechanisms. In this context, it is noteworthy that the predominant types of cutaneous malignancies in XP are melanoma, squamous cell carcinoma and basal cell carcinoma, the advanced forms of which are responsive to monoclonal antibody-based, mostly PD-1-targeted, immunotherapy (58–61). However, given the findings of the current study, the co-existence of several types of systemic, soluble inhibitory checkpoints in XP patients suggests that combination therapy targeting additional immune checkpoints may be most effective, despite the increased risk of development of immune-related adverse events (irAEs). In addition, future studies should also focus on associations between systemic soluble inhibitory immune checkpoints and the expression levels of these molecules on tumor cells and other types of cell in the TME. Furthermore, additional work investigating the prognostic and predictive potential of plasma levels of soluble inhibitory immune checkpoints such as PD-L1 should be further researched in XP. An additional point of interest is the existence of a relationship between high plasma levels of soluble PD-L1 and CTLA-4 with the possible lack of efficacy of monoclonal antibody-based checkpoint-targeted immunotherapy, which should also be evaluated in XP (40).

The frequency of low plasma levels of vitamin D detected in many XP patients in the current study, which is probably due to strict implementation of photo-protective measures, is of potential significance for several reasons. Firstly, the apparent anti-cancer properties of the vitamin (26, 27) and, secondly, its potential clinical utility, in reducing the severity of irAEs associated with checkpoint-targeted immunotherapy of melanoma, possibly by regulating the pro-inflammatory activity of Th17 cells, which have been implicated in the pathogenesis of some types of irAE (62–64). In another recent study, however, deficiency of vitamin D, as opposed to supplementation with the vitamin, was not associated with an increased frequency of irAEs in patients with various types of cancer (65).

Finally, taking into consideration that XP is a rare disease, the limitations of the current study include a relatively small, albeit unavoidable, sample size and difficulty in recruiting closely age-matched controls due to the very young ages of many of the patients, as well as measurement of the test circulating biomarkers at a single time point, as opposed to over an extended period. Irrespective of these issues, however, the findings of the current study have highlighted the existence of major upregulation of negative immune checkpoint pathways that may contribute to poor pre-cancer immune surveillance, immune evasion of pre-malignant and malignant cells and generalized tumorigenesis in South African XP patients.

## Conclusions

The results of this study have revealed the existence of chronic systemic inflammation and immunosuppression in patients with XP. Whether these are simply secondary to their primary disease and coexistent malignancies, or whether they contribute to the development and spread of cancer in XP participants remains to be established. In this context, the finding of substantially increased plasma levels of several inhibitory immune checkpoint proteins, an occurrence that is probably secondary to the high prevalence of the various types of advanced cutaneous malignancies associated with XP, is seemingly of greatest significance. Although speculative, the low levels of vitamin D detected in the plasma of a high percentage of XP patients may also contribute to immune dysfunction and tumorigenesis. Future studies exploring possible relationships between the composite tumor burden, types and concentrations of plasma immune checkpoints, efficacy of specific checkpoint-targeted immunotherapy (combination therapy?) and levels of vitamin D, may provide improved insight regarding the pathogenetic role of immune dysfunction/inflammation in XP. This, in turn, may contribute to better management interventions through discerning exploration of novel immune/inflammatory-targeted therapies. Follow-up studies would also benefit from implementation of extensive collaborations spanning various geographic regions, enabling access to larger cohorts of XP patients. Finally, measurement of systemic levels of soluble PD-L1, PD1 and CTLA-4 may be of value in the selection of patients with skin cancer for immune checkpoint inhibitor treatment.

## Data Availability Statement

The raw data supporting the conclusions of this article will be made available by the authors, without undue reservation.

## Ethics Statement

The studies involving human participants were reviewed and approved by the Research Ethics Committee of the Faculty of Health Sciences, University of Pretoria. Written informed consent to participate in this study was provided by the participants’ legal guardian/next of kin.

## Author Contributions

All authors contributed significantly to the conceptualization and planning of the manuscript. MK and KA provided clinical oversight, while HS, LK, KA and SS performed the laboratory investigations. PM advised on biostatistical issues. All authors contributed to analysis and interpretation of data, as well as to the preparation of the manuscript and all consented to submission of the final version.

## Funding

The study was supported by the University of Pretoria.

## Conflict of Interest

The authors declare that the research was conducted in the absence of any commercial or financial relationships that could be construed as a potential conflict of interest.

## Publisher’s Note

All claims expressed in this article are solely those of the authors and do not necessarily represent those of their affiliated organizations, or those of the publisher, the editors and the reviewers. Any product that may be evaluated in this article, or claim that may be made by its manufacturer, is not guaranteed or endorsed by the publisher.
